# AI-accelerated meta-analysis in psychology: Large language models code study properties with high accuracy

**DOI:** 10.3758/s13428-026-03020-1

**Published:** 2026-04-27

**Authors:** Shaheed Azaad

**Affiliations:** Institute of Psychology, University of Münster, Münster, Germany

**Keywords:** Large language models, Artificial intelligence, Meta-analysis, Systematic review, Study coding

## Abstract

Theoretically driven meta-analyses often involve testing whether study characteristics moderate an effect in a pattern that supports one of multiple competing theoretical accounts. To conduct such analyses, researchers must manually extract, i.e., code, these characteristics from sometimes hundreds of studies. Ideally, studies should be coded by two researchers independently to prevent errors and biases from compromising the dataset’s integrity. The laborious nature of this task, however, means that meta-analysts usually settle for double-coding just a portion of the studies included in their study. Some researchers have proposed using large language models (LLMs) as a double-coder, showing that they can reliably extract explicitly stated information (e.g., publication year) from articles. However, meta-analyses in psychology generally require studies to be coded in terms of higher-level conceptual dimensions (e.g., the type of behavior rather than which behavior). The present study investigated whether two LLMs, OpenAI’s GPT-5 and Google’s Gemini 2.5 Pro, can code studies in this way. Both models replicated the study codes from three recently published meta-analyses in psychology with high accuracy (>92% overall), despite the limited information they received. LLM double-coding, therefore, offers a practical solution for meta-analysts seeking to ensure data integrity.

**Supplementary Information:**

The online version contains supplementary material available at 10.3758/s13428-026-03020-1.

## Introduction

Meta-analysis enables research psychologists to test for a phenomenon across multiple studies (Borenstein, 2009). In addition to computing a summary statistic, larger meta-analyses also test hypotheses derived from competing accounts of the phenomenon of interest (e.g., Azaad et al., 2019; Ingendahl et al., 2025). They do so by investigating whether study properties moderate an effect as predicted by one theory versus another. Obtaining data for such analyses requires meta-analysts to read and manually extract information about, or *code*, these study properties for sometimes hundreds of studies. Ideally, a second researcher will independently double-code these studies, minimizing potential errors and biases associated with manual data extraction (Barchard & Pace, 2011). However, the laborious nature of this critical step means that authors of even high-quality meta-analyses will often double-code only a portion of all included studies (e.g., Ingendahl et al., 2025).

Failing to double-code can compromise the integrity of a meta-analysis’s results. Manual data entry is strikingly error-prone, and mere visual inspection is insufficient to detect these errors (Barchard & Pace, 2011). Even if researchers somehow avoided making any mistakes in data extraction and entry, this would only ensure data integrity for clear and objective study characteristics (e.g., the mean age of a sample). In psychology, meta-analysts often categorize studies based on their conceptual properties. This process involves decision-making based on pre-defined criteria. For instance, in a recent study, Wu et al. (2025) meta-analyzed the relationship between social class and prosociality. Included studies used a range of prosociality measures. Rather than creating separate moderator categories for each measure, the authors coded them conceptually, for example, distinguishing between studies that measured prosocial intentions and those that measured behaviors. Such coding based on conceptual properties is susceptible to differences in interpretation, edge cases, and researcher degrees of freedom. Independently double-coding all included studies would go some way toward mitigating these risks. Given a recent simulation study showing that miscategorizing just 2% of moderator codes can substantially affect discovery rates (Azaad & Friebe, 2025), the fact that many meta-analyses fail to do so means their results may remain open to question.

One solution would be to require that authors double-code all studies in their dataset. However, this could be prohibitively burdensome for many researchers seeking to synthesize large bodies of empirical literature in detail. Recent advancements in artificial intelligence (AI) present a more compelling alternative – double coding using LLMs. Researchers have already started adopting AI tools for study screening and data extraction (Blaizot et al., 2022; van Dijk et al., 2023). There is limited data on how accurately these tools can extract study information with the detail and precision required for meta-analysis. The few studies that have explored whether LLMs can code studies have used them to code simple, objective study characteristics such as the publication year, sample demographics, and aspects of the method that authors would report explicitly, such as the treatment dose in a clinical trial (Gartlehner et al., 2024; Helms Andersen et al., 2025). As a result, these studies tell us little about whether LLMs can code studies along conceptually relevant dimensions in a way that enables psychologists to conduct theory-driven meta-analyses.

Reliable and accurate AI study coding could benefit meta-analytic research synthesis beyond merely ensuring data integrity. Meta-analysts may be encouraged to take a more comprehensive approach by coding for a broader range of moderators or including more studies (e.g., by expanding the date range of their literature search), since the time cost for doing so would be trivial. Importantly, AI study coding would improve the replicability and transparency of study coding. Researchers would be able to share their AI configurations and instructions alongside their published meta-analyses, enabling others to recreate their datasets and, therefore, their analyses with high reliability.

Given these potential advantages, and that meta-analyses play a critical role in theory testing in psychology, an investigation into AI’s capacity to code study characteristics at a conceptual level is due. To this end, the present study tested the accuracy with which two flagship LLMs – OpenAI’s GPT-5 and Google’s Gemini 2.5 Pro – could replicate study codes reported from three recent, high-quality, meta-analyses in psychology.

## Method

### Meta-analysis selection

I manually searched for articles, starting with the most recent publications in *Psychological Bulletin*, until I identified meta-analyses suitable for LLM coding. The search spanned five issues of Volume 161 (Issues 3–7) and included 18 articles. Articles were ineligible if: they did not contain a meta-analysis (*n =* 1), they did not contain a moderator analysis (*n* = 2), they contained a re-analysis of data gathered by others (*n* =5), they analyzed fewer than five moderators (*n* = 1), they did not contain a moderator table or coding instructions in either the main text or supplementary materials (*n* = 4), their data were unavailable online (*n* = 1), their data did not come with enough information (e.g., keys and notes) to compare codes (*n* = 1).

This search yielded the following three meta-analyses (Table 1 for overview):

**Table 1:** Overview of included meta-analyses

Study	Description of focal phenomenon	Example conceptual moderator	Journals (*n* articles)	Reported interrater agreement (IRA)
Ingendahl et al.	Learners’ memory for studied materials is affected by making judgments about their learning (estimated future performance) immediately after study	Type of memory test: the type of memory test used as an outcome measure (e.g., cued recall or recognition)	Memory & Cognition (6) Journal of Intelligence (4) Metacognition and Learning (3) Journal of Experimental Psychology Learning, Memory, and Cognition (2) Psychonomic Bulletin & Review (2) Journal of Applied Research in Memory and Cognition (1) Journal of Memory and Language (1) Memory (1)	IRA was 94%; 20% of experiments were double-coded
Shakeri and North	The convergence of attitudes toward older (versus younger) men and women	Attitude dimension: the type of attitude for which a study probed (e.g., attractiveness or warmth)	The International Journal of Aging and Human Development (3) Anatomical Sciences Education (2) Ageing and Society (1) Educational Gerontology (1) European Journal of Ageing (1) European Journal of Psychological Assessment (1) Human Relations (1) Journal of Adult Development (1) Journal of Business and Psychology (1) Journal of Management & Organization (1) Journal of Political Economy (1) PLOS One (1) The Journal of Social Psychology (1) Current Research in Social Psychology (1) Personality and Social Psychology Bulletin (1)	Authors double-coded two moderators for 27% of sources, achieving Cohen’s κs of 1 and.80, respectively
Wu et al.	The relationship between social class and prosociality	Prosociality measure: whether the study measured actual prosocial behaviors or just the intention to act prosocially	VOLUNTAS International Journal of Voluntary and Nonprofit Organizations (2) British Journal of Social Psychology (1) Cognition and Emotion (1) Cognitive Development (1) Current Psychology (1) Infancy (1) International Journal of Environmental Research and Public Health (1) Journal of Child and Family Studies (1) Journal of Community & Applied Social Psychology (1) Journal of Experimental Child Psychology (1) PLOS One (1) Rural Sociology (1) Social Development (1) The Journal of Sociology & Social Welfare (1) Journal of Black Psychology (1) The International Journal of Volunteer Administration (1) Journal of Applied Social Psychology (1)	All studies were double-coded. Krippendorff’s αs were between.72 and 1. The lowest IRA was 87%

1. Ingendahl et al. (2025) - Do immediate judgments of learning alter memory performance? A meta-analytical review.
2. Shakeri and North (2025) - The Gender Convergence Effect in Older Age: A Meta-Analytic Review Comparing Modern Attitudes Toward Younger, Middle-Aged, and Older Women and Men
3. Wu et al. (2025) - Social Class and Prosociality: A Meta-Analytic Review

### Source selection

I randomly selected 20 published studies from each article for LLM coding (for Wu et al., I filtered out studies not in English). Some sources contributed multiple studies to the dataset. To prevent these from biasing estimates of reliability, I included only one study per source, resulting in a coding dataset containing 20 unique sources. I provided the LLMs with identifying information from the datasets (e.g., study numbers, outcome measures, conditions) to indicate which study to code from multi-study sources.

### LLM coding

I coded articles using two models: OpenAI’s GPT-5 and Google’s Gemini 2.5 Pro through their respective Node.js packages (version 5.23.1 for GPT-5 and 1.21.0 for Gemini). For Gemini, I set the temperature – or the randomness in the model’s responses – to zero. This option was unavailable for GPT-5^1^. To ensure that the models returned data in a consistent format that could be immediately collated and analyzed, I configured both models to return data in JavaScript Object Notation format rather than chatbot-style responses.

For each meta-analysis, I provided the models with (1) system instructions, which included general guidelines for extracting study characteristics, (2) a coding manual that described the meta-analytic moderators, (3) source articles, and (4) data templates for the model to fill with codes. System instructions emphasized that the model should not use external information to code articles and should adhere to the provided coding manual. These instructions were the same for all meta-analyses, except where I added minor prompts to ensure consistency in formatting for continuous codes (e.g., to report proportions instead of percentages). The coding manual included moderator tables and more detailed descriptions of moderators from the articles’ main text or supplementary materials. I made minor edits to content from the original article to ensure consistency (e.g., moderator and level names) between the coding manual and the published dataset.

I instructed models to provide a brief explanation for their codes, asking them to quote the article or provide a page reference where possible. This enabled checks to ensure that models did not use external information to code articles, and facilitated investigations into systematic coding discrepancies. Minor edits were made to generated codes to ensure consistency where coding materials did not specify precisely how a code should be formatted (e.g., whether “United States” should be coded as “US”). Edits were also made to the original authors’ code to address inconsistencies in punctuation or spelling.

## Results

For each meta-analysis, I computed interrater agreement (IRA) between LLM-generated and author study codes for all study characteristics included in the moderator analyses. This was computed using the *irr* (Gamer et al., 2005) package for *R* (R Core Team, 2013)*.*

### Ingendahl et al.

GPT-5 achieved a high average percentage agreement of 93% (SD = 13, min = 60%, max = 100%). Gemini 2.5 Pro’s accuracy was lower, albeit still high, achieving a mean of 90% (SD = 6.9, min = 60%, max = 100%). Percentage agreements between the two LLMs were higher (96%, SD = 7, min = 80%, max =100%), indicating that discrepancies may have been systematic. Inspection of IRAs by moderators revealed that the two continuous moderators accounted for most discrepancies (80% for GPT-5 and 60% for Gemini 2.5 Pro).

Correspondence with the lead author revealed that codes for these variables were often derived from raw data or obtained by emailing the source authors. In many cases, it was therefore not possible for the models to obtain this data from the provided information. Excluding these two moderators substantially boosted IRAs; GPT-5 achieved an agreement of 98% (SD = 3, min = 95%, max = 100%), while Gemini 2.5 Pro achieved 95% (SD = 6, min = 85%, max = 100%). All three remaining discrepancies between GPT-5 and the authors were also shared with Gemini 2.5 Pro, suggesting that they reflect ambiguity in the source articles or the coding materials.

### Shakeri and North

An initial set of LLM-generated codes for Shakeri and North revealed ambiguities in the coding manual, resulting in systematic discrepancies across both models. Additionally, as in Ingendahl et al., the authors relied heavily on raw data for one moderator. For the analyses reported here, I excluded these moderators and amended the coding manual after corresponding with the lead author. I detail this process and the results from the first round of coding in the Supplementary Materials.

The interrater agreements reported here are based on a new set of codes generated from a second, independent sample of randomly selected sources in Shakeri and North’s dataset. These codes were for moderators that could be reliably coded from the articles alone. Gemini 2.5 Pro achieved 89% agreement (SD = 8, min = 75%, max = 100%) with Shakeri and North’s codes, whereas GPT-5 achieved 90% agreement (SD = 8, min = 70%, max =100%). Agreement between the two models was again higher at 94% (SD = 6, min = 85%, max = 100%). Of the 29 total disagreements between the models and Shakeri and North’s codes, 62% were common to both models.

### Wu et al.

Both models achieved 93% agreement with Wu et al.’s codes (GPT-5: 93%, SD = 7, min = 85%, max = 100%; Gemini 2.5 Pro: 93%, SD = 6, min = 80%, max = 100%). Agreement between the models was only slightly higher at 94% (SD = 7, min = 80%, max = 100%). Of the total disagreements, 38% were common to both models. None of Wu et al.’s moderators appeared to be coded from information not reported in the source articles.

## Summary

**Fig. 1:**
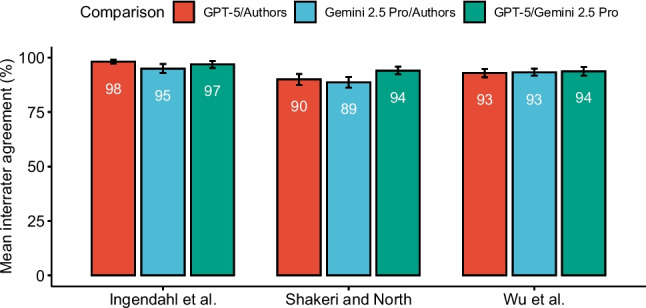
Interrater agreements across meta-analyses. *Note*: Interrater agreement for Ingendahl et al. excludes two moderators that could not be coded reliably from source articles due to missing information

**Fig. 2:**
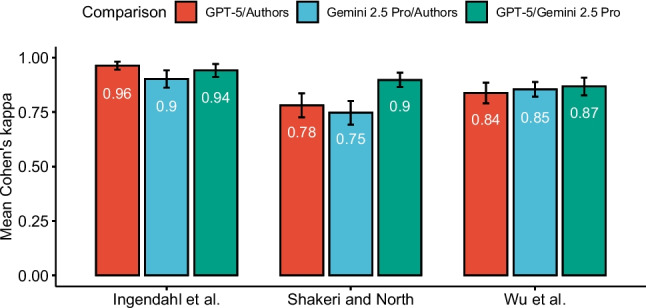
Cohen’s kappa across meta-analyses. *Note*: Kappa values for Ingendahl et al. exclude two moderators that could not be coded reliably from source articles due to missing information.

Overall, the two LLMs achieved similar accuracy across all three meta-analyses for moderators that could be coded directly from the source articles (Fig. 1). GPT-5’s overall accuracy was 93% (SD = 7, min = 70%, max = 100%) while Gemini 2.5 Pro achieved 92% (SD = 7, min = 75%, max = 100%). A limitation of using IRA as a measure of agreement is that it does not tell us to what extent this agreement exceeded the chance level. To address this, I computed Cohen’s kappa (κ), which ranges from – 1 to 1, with values above 0 indicating beyond-chance agreement (Fig. 2). Values above.41,.61, and.81 are generally interpreted as moderate, substantial, and almost perfect, respectively (Landis & Koch, 1977). Both models achieved almost perfect agreement for Ingendahl et al. (Gemini 2.5 Pro κ =.90; GPT-5 κ =.96), and Wu et al. (Gemini 2.5 Pro κ =.86; GPT-5 κ =.84). Agreement with Shakeri et al.’s codes was substantial (Gemini 2.5 Pro κ =.75; GPT-5 κ =.78).

## Discussion

Meta-analysis is a powerful tool not only for summarizing a body of literature but also for conducting theoretically driven tests of moderation. The validity of these tests depends on researchers accurately coding the characteristics of the studies included in their meta-analysis. Despite the risk that errors (Barchard & Pace, 2011) and biases may compromise this process, the laborious nature of study coding means that meta-analysts often only double-code a fraction of the studies they include. Here, I demonstrate that LLMs may be a viable alternative to human double coders. Gemini 2.5 Pro and GPT-5 were able to replicate the codes of three recently published meta-analyses with an accuracy of over 92%.

The present study demonstrates that, in addition to extracting objective and explicitly reported study properties (e.g., publication year), LLMs can code study properties at a conceptual level—a necessity for meta-analytic theory testing. Another novel aspect of this study is that it generated structured datasets programmatically rather than using chat interfaces (Gartlehner et al., 2024; Helms Andersen et al., 2025) to solicit codes from LLMs. Using chat interfaces fails to address some of the problems of manual coding in the first place. Data entry errors remain possible because researchers must manually transfer LLM responses into their dataset. Moreover, manually converting conversational text responses into data for analysis may still be subject to bias or differences in interpretation.

A very recent study by Jansen et al. (2025), published shortly before the present study was submitted for review, found that LLMs coded study properties (excluding effect sizes, which I did not include in this study) with an accuracy of just 69%, with the best model – Claude 3 Sonnet – achieving 75% accuracy. An obvious explanation for this discrepancy is that the models Jansen et al. used were older than the ones I tested here. While Jansen et al. did not test a previous version of Gemini, they did report that GPT-4o (GPT-5’s predecessor) achieved an overall accuracy of 71% of study properties. Given that Jansen et al. found state-of-the-art *frontier* models coded most accurately, it seems that less advanced models are more likely to make coding errors. This would explain why Gemini 2.5 Pro and GPT-5, which are substantially newer than the models included in Jansen et al.’s study, demonstrated higher accuracy.

The potential benefits of using AI to double-code are several and significant. Not only would it significantly accelerate the coding process, but it would also encourage meta-analysts to double-code their complete set of studies. This, in turn, would improve the quality of meta-analytic datasets and enhance the veracity of their results. Furthermore, the time and effort saved may lead researchers to conduct meta-analyses more often, with larger sets of studies and a broader range of moderators. Finally, AI-coding would go some way toward making the coding process more transparent. This is partly because it would require researchers to create highly detailed, comprehensive coding materials, reducing their own degrees of freedom during coding. Further, sharing AI configurations alongside coding instructions would improve the transparency of the coding process by enabling researchers to replicate the authors’ codes with minimal effort.

One limitation of the present study concerns its generalizability. On the one hand, I examined whether the models could reproduce codes for only three meta-analyses. While they covered different topics, suggesting some generalizability, there may be disciplines in which LLMs fail to code accurately. However, given that Jansen et al. (2025) did not find evidence that the meta-analysis discipline moderates code accuracy, instead finding that coding accuracy depended on the availability of coding materials, this seems unlikely. Moreover, although I included only three meta-analyses, the coded source articles spanned more than 30 journals (Table 1), suggesting that our results generalize across different publication styles. Another limitation is that I generated codes using only two models. Given that frontier models tend to outperform others (Jansen et al., 2025), GPT-5 and Gemini 2.5 Pro might represent the current (at the time of this study) best-case scenario for LLM coding. However, this is likely to change as substantial advances in LLM quality are made. Nevertheless, larger-scale studies are needed to confirm the generalizability of the present results.

Other limitations would have likely increased, rather than decreased, the LLMs’ accuracy. For one, operationalizing accuracy as IRA assumes that the authors of the included meta-analyses always entered the correct codes. Considering that many of the authors’ entries across the three meta-analyses were not double-coded, it is highly unlikely that they were entirely error-free. So, there may have been instances in which the LLMs indeed extracted the correct code, whereas the authors did not. This would also explain why IRRs were lowest for Shakeri and North, who double-coded the fewest studies across the three meta-analyses (Table 1). Moreover, although I excluded some moderators for which the authors relied on information not reported in source articles, there may still have been codes in the remaining moderators that came from external information.

Lastly, the coding instructions that models received were basic descriptions of moderators that were not designed to be interpreted and used by a naïve reader. Ideally, coding instructions would be unambiguous, include guidance on handling edge cases, and specify when and whether the coder should draw inferences or make assumptions. Inspection of study codes revealed a few instances where a clearly defined coding manual would have increased the models’ accuracy. For example, Shakeri and North (2025) drew a dichotomy between ‘work’ and ‘non-work’ contexts in their meta-analysis; however, whereas the authors coded contexts such as an election as a ‘work’ context, the models coded them as being ‘non-work’. Constructing a comprehensive coding manual that provides guidance on such cases would likely have improved the alignment between the models’ and authors’ codes. For these reasons, this study likely *underestimates* the accuracy with which LLMs can perform meta-analytic coding.

A strength of this study is that it uses published meta-analyses to ensure the ecological validity of the models’ observed accuracies. However, this leaves open the possibility that the models may have found the authors’ codes online and reported them as their own. This is highly unlikely for several reasons. One, I did not provide any information about the meta-analyses to the models. Therefore, it is unlikely that the models had sufficient information to locate the authors’ datasets online, even if they had sought them. Two, the inspection of models’ explanations shows that the models accurately quoted the text on which they based their provided codes. Finally, LLM codes were systematically less accurate for codes with ambiguous instructions or missing data in the main text.

In all, LLMs appear capable of serving as efficient and reliable double coders for meta-analyses in psychology and are likely more accurate than the present study suggests. Results from this study and from Jansen et al. (2025) suggest that using the most advanced models available and providing them with detailed coding instructions yields the most accurate codes. Using LLMs for meta-analysis has the potential to improve the quality, efficiency, scope, and transparency of meta-analytic synthesis.

## Supplementary information

Below is the link to the electronic supplementary material.

Supplementary file1 (PDF 54.2 KB)

## Data Availability

Data and analysis scripts are available at https://osf.io/b89xs/.

## Footnotes

1 To test the variability of codes generated by GPT-5, I generated a second set of codes around 3 months after the original set. Percentage agreements between the two sets were high (96%, SD = 5, min = 85%, max = 100%). Interestingly, the three codes with the lowest agreement between sets, at 85%, also had a percentage agreement of 85% with the study authors’ codes in the first sets. This might suggest that disagreement between sets were due to variability in the models’ handling of ambiguity in either the coding materials or source articles.

## Code availability

Relevant code is available at https://osf.io/b89xs/.
