# Supplementary material for: AI-accelerated meta-analysis in psychology: Large language models code study properties with high accuracy
Source: Behav Res Methods. 2026 Apr 27;58(6):143. doi: 10.3758/s13428-026-03020-1 (PMC13121511; doi:10.3758/s13428-026-03020-1)
Supplement: Supplementary file 1 — Supplementary file1 (PDF 54.2 KB) [file 13428_2026_3020_MOESM1_ESM.pdf]

Supplementary materials

S1. Interrater agreements for Shakeri and North prior to coding manual revisions

Figure S1. Interrater agreements for Shakeri and North prior to coding manual revisions

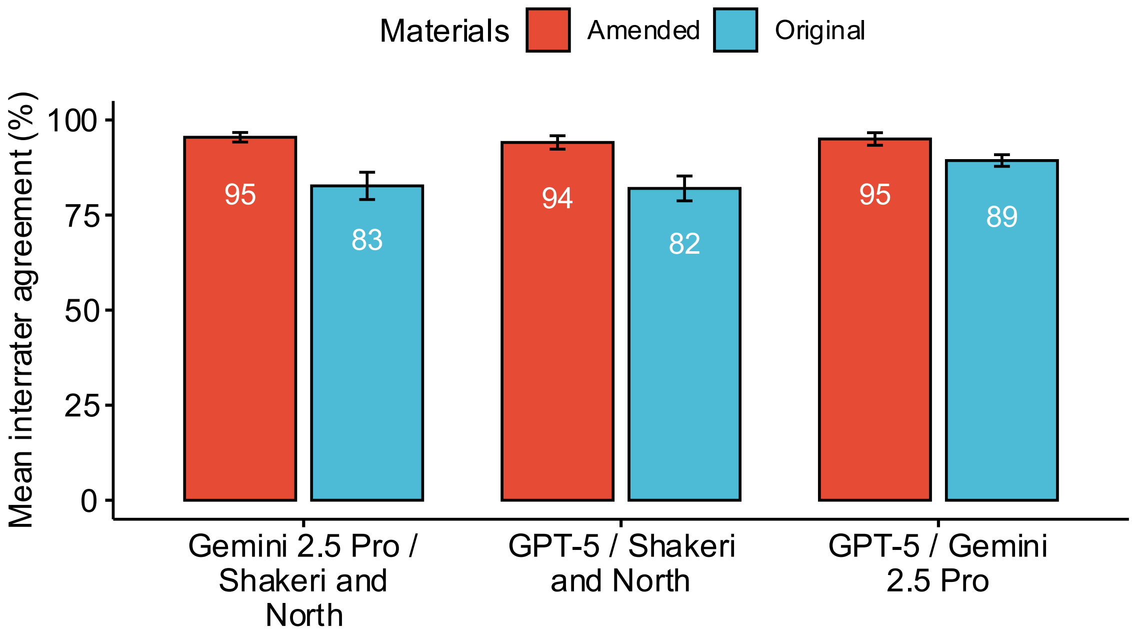

Prior to revising the coding manual for Shakeri and North, interrater reliabilities for both GPT-5 (M = 82%, SD = 13) and Gemini 2.5 Pro (M = 83%, SD = 14) were low (Figure S1). Of the original codes that had discrepancies with at least one model, 60% were discrepant with codes from both, implying ambiguity in the coding manual. Inspection of discrepant codes revealed that a substantial proportion of these came from a handful of moderators. These reflected two problems. The first was that demographic information (e.g., the percentage of male participants) was often computed from raw data and was therefore not available to the models, which only had access to articles. The second issue was ambiguity in Shakeri and North's code descriptions. For example, the authors drew a dichotomy between lab and field studies in their meta-analysis, but did not elaborate on how they decided to place studies into these categories.

For this moderator, the models coded online studies as being field studies, whereas the authors coded them as being lab studies.

After confirming these issues by corresponding with the meta-analysis's lead author (Shakeri), I revised the coding procedure to obtain a set of codes more representative of the models' capacity. This involved dropping demographic moderators and revising the coding material by (1) adding text to resolve major and obvious ambiguities in Shakeri and North's coding table, and (2) adding text from Shakeri and North's published meta-analysis, which elaborated on their coding strategy for two moderators. After making these changes, I generated a new set of codes. These changes increased the accuracy of both models (Figure S1) to over 94%. Nevertheless, a substantial portion of the remaining disagreements were still common to both models (35%), suggesting residual ambiguity in the coding materials. These reliabilities were higher than those reported in the main text, which are for a new set of articles. This is likely due to new, unaccounted-for ambiguities or edge cases that did not appear in the original set of codes.
